# Pediatric Autoimmune Neuropsychiatric Disorder Linked to Streptococcal Infections

**DOI:** 10.1155/2023/6667272

**Published:** 2023-12-11

**Authors:** Abdullah Nasser Alqifari, Benjamin Maxwell

**Affiliations:** ^1^Department of Psychiatry, College of Medicine, Qassim University, Buraydah, Saudi Arabia; ^2^Rady Children's Hospital of San Diego, San Diego, CA, USA; ^3^Department of Psychiatry, University of California San Diego, La Jolla, San Diego, CA, USA

## Abstract

Pediatric acute-onset neuropsychiatric syndrome (PANS) is a clinical condition with abrupt onset of obsessive–compulsive symptoms and/or severe eating restrictions and at least two concomitant cognitive, behavioral, or neurological symptoms. Pediatric autoimmune neuropsychiatric disorder associated with streptococcal infections (PANDAS) is a subtype of PANS with a controversial diagnosis. A case of a 12-year-old girl with severe eating restriction, obsessive–compulsive symptoms, anxiety, and neurological symptoms who initially was diagnosed with obsessive–compulsive disorder is presented. Published reports were reviewed for the evidence of diagnosis and treatment options of PANS/PANDAS. Studies show controversy regarding diagnosis. Moreover, study reports showed limited evidence for the treatment options. Selective serotonin reuptake inhibitors and psychotherapy are considered the main treatment with prompt infection treatment in the case of PANDAS.

## 1. Introduction

Pediatric acute-onset neuropsychiatric syndrome (PANS) is a clinical condition with abrupt onset of obsessive–compulsive symptoms and/or severe eating restrictions and at least two concomitant cognitive, behavioral, or neurological symptoms. Pediatric autoimmune neuropsychiatric disorder associated with streptococcal infections (PANDAS) is a subset of PANS. PANS and PANDAS are emerging autoimmune encephalopathies of childhood [1].

The incidence and prevalence of PANDAS are unknown, although they are considered rare [2, 3]. According to the PANDAS network, it has been postulated that the prevalence of this disorder would be higher than previously acknowledged, potentially impacting up to 1 in 200 children [3]. In 2021, the Pediatric Acute-Onset Neuropsychiatric Syndrome Advisory Council in Texas released a report with data concerning age and gender demographics [3]. According to the findings, most children diagnosed with PANS are often observed between the age range of 1–13 years. Specifically, over 60% of diagnoses occur between the ages of 4 and 9 years, with the highest frequency of diagnosis observed at roughly 6.5 years of age. The male population exceeds the female population by a ratio of around 2 to 1 [3]. Furthermore, it was reported that there is variability in the prevalence of PANS among patients with different disorders. Specifically, it was reported that the prevalence of PANS/PANDAS among pediatric with tic disorder to be 11%. However, the prevalence is much lower among pediatric obsessive–compulsive disorder (OCD) in outpatient and children at a movement disorder clinic, which was 5% and 1%, respectively. Among eating disorders, it was reported that the prevalence of PANS is 52% and 0% for PANDAS [4].

Several treatment options have been proposed for PANS/PANDAS. Still, no clear therapeutic protocol has been recognized to prevent these neuropsychiatric diseases [5].

PANDASs have attracted much interest and discussion since it was originally characterized in 1998. The role of streptococcal infection in children with abrupt-onset OCD and new-onset tics, the natural history of this entity, and the role of symptomatic and disease-modifying therapies, such as antibiotics, immunotherapy, and psychoactive drugs, are still unresolved issues [6].

This case report presents an uncommon yet interesting neuropsychiatric case and discusses the controversies in the diagnosis and management.

## 2. Case Presentation

A 12-year-old girl presented with an acute onset of a 1month history of recurrent undesired and intrusive thoughts demanding her to repeat or stop doing certain behaviors to prevent unrelated danger (killing herself or going out in the street). She has stopped eating. The family frequently visited the emergency room (ER) to administer intravenous (IV) fluids. She started dressing and redressing for around 1 hr, repeated washing for around 1 hr, crossing her hands and scratching the wall, and crying and fearing killing herself or going out in the street. Family history: living with her mother. Her father passed away 2 years before presentation due to a medical illness. There is no history of mental illness in the family. She was doing well in school but had frequent absences in the last month before the presentation. Pregnancy, birth, and early development are unremarkable. Medical history: in this initial visit, the family denied the patient had any medical illnesses. The impression was that the patient had OCD. The treatment was SSRI and cognitive-behavioral therapy (CBT). However, because of the acute onset and severity of her symptoms, the patient was reevaluated in follow-up with consideration of possible pediatric acute neuropsychiatric syndrome. She reported that she had recurrent tonsillitis in the last year before presentation, and her last infection was just before the start of her symptoms. Her recurrent tonsillitis was severe to the extent that a tonsillectomy was advised 6-month ago. The obsessive thoughts were associated with symptoms of separation anxiety, being forgetful, and abnormal sensations (tingling sensation) in her palms and feet. She repeats wearing her shoes because of these abnormal sensations. The assessment was that most likely she has PANDAS as she has recurrent tonsilitis and met consensus PANS criteria [7] by having:

(1) Abrupt, dramatic onset of OCD or severely restricted food intake
(2) Concurrent presence of additional neuropsychiatric symptoms:
  - Anxiety
  - Sensory abnormalities
(3) Symptoms are not better explained by a known neurologic or medical disorder, such as Sydenham chorea (SC).

Other differential diagnoses were considered, including OCD and prodromal stage of psychotic illness.

Initial investigations were requested, which were complete blood count (CBC) with differential, C-reactive protein (CRP), erythrocyte sedimentation rate (ESR), antistreptolysin O (ASO), and throat culture. Lab results showed ASO = around 1,200 (normal < 200).

Admission to the hospital was requested because of the restricted eating and for further evaluation and workup to rule out other causes and to start antibiotics by pediatrics if needed.

The patient stayed in the hospital for 2 days. During hospitalization, the patient was seen by the pediatric team, and they ruled out other medical causes and complications. Medical history and physical examination, including neurological examination with workup including systemic lupus erythematosus (SLE) markers and cardiac echo, were done. All were reassuring. Throat culture results came out, which showed mixed normal flora (however, lab error was assumed by the pediatric team).

For treatment intervention, an antibiotic was started empirically to eradicate the infection. The ER started Ampicillin 1 g IV four times a day (QID) on the night of admission. This was shifted the next day to Cefuroxime 1 g IV three times per day (q8 hr) because it seemed most suitable per the pediatric team. SSRI was continued. The patient dramatically improved eating the next day after starting the antibiotic. She was discharged after 2 days with follow-up with psychiatric and pediatric clinic. She still had OCD symptoms, thoughts that she may kill herself or go out to the street, and neurological symptoms.

Consideration of other treatment options was discussed. Options were nonsteroidal anti-inflammatory drugs (NSAIDs), steroids, tonsillectomy, and intravenous immunoglobulin (IVIG). A trial of steroids was started. The plan was to increase the dose to 60 mg/daily. When it reached 30 mg/daily within 3 days, the result was a minimum response with decreased crying episodes and a slight decrease in suicidal thoughts. However, the family mentioned that she had developed thoughts about killing her mother. Moreover, she developed a sore throat. To avoid this complication, the initial plan was to start the steroid while she was on antibiotics, but she finished the antibiotic course when the steroid started. The steroid was stopped.

A trial of increasing SSRI was tried. Fluoxetine was gradually increased from 20 mg daily to 40 mg daily. With the increased SSRI dose, she relapsed by not eating and being anxious. The family decreased the dose by themselves, and she resumed her previous state of eating.

On the last visit, abnormal sensations at the hand and feet were better but still there. The OCD symptoms were still the same, but anxiety was better. She has resumed going to school. Her sleep and eating were within normal. She was still having recurrent pharyngitis frequently.

## 3. Discussion

The presented case meets the working criteria for PANS that were prepared by a panel of experts in July 2010 at the National Institutes of Health (NIH). The prepared consensus PANS criteria were as follows: [7]

(i) Abrupt, dramatic onset of OCD or severely restricted food intake [7, 8].
(ii) Concurrent presence of additional neuropsychiatric symptoms (with similarly severe and acute onset) from at least two of the following seven categories:
  1. Anxiety
  2. Emotional lability and/or depression
  3. Irritability, aggression, and/or severely oppositional behaviors
  4. Behavioral (developmental) regression
  5. Deterioration in school performance (related to attention-deficit/hyperactivity disorder- (ADHD-)like symptoms, memory deficits, cognitive changes)
  6. Sensory or motor abnormalities
  7. Somatic signs and symptoms, including sleep disturbances, enuresis, or urinary frequency
(iii) Symptoms are not better explained by a known neurologic or medical disorder, such as SC.

One of the criteria of PANS is the presence of OCD. Although OCD is a clinical diagnosis, additional evaluation may involve the application of the Yale-Brown Obsessive–Compulsive Scale. The Yale-Brown Obsessive–Compulsive Scale is the gold standard for evaluating the symptoms of OCD [9–13]. The Y-BOCS is a clinician-administered, semistructured interview that is frequently utilized in clinical and research settings to assess the severity of symptoms in patients who have been diagnosed with OCD [9–13]. While the instrument was not used in the case presented, the author suggests applying it if feasible for such cases. PANS has an extensive array of potential differential diagnoses that need thorough screening and evaluation. Differential diagnoses for youth with PANS include OCD, anorexia nervosa, avoidant/restrictive food intake disorder, tourette syndrome, transient tic disorder, bipolar disorder, Sydenham chorea, autoimmune encephalitis, systemic autoimmune disease, and Wilson disease [7]. It was recommended to screen all patients meeting criteria for PANS by CBC, ESR, CRP, metabolic panel, urinalysis, throat culture, antistreptolysin O, and anti-DNAse B. Additional investigations that need consideration include antinuclear antibodies (ANA), antiphospholipid antibodies (APA), and ceruloplasmin and 24 urine copper tests [7].

It was reported that the evaluation of PANS should consider infectious disease, immunodeficiency, and autoimmune disease. Infectious disease in a patient with PANS, such as the case presented, may indicate a diagnosis of PANDAS, a subtype of PANS, that is distinguished by a history of recurrent infections confirmed by rapid antigen testing (RAT), culture, and ASO [7]. Immunodeficiency should be considered if PANS criteria are met with repeated infections, infection with atypical organism, and if there is a family history of first degree with fatal infection. An initial workup may include lymphocyte subsets, quantitative immunoglobins, and vaccine responses [7]. In the current case, the patient's prior antibiotic type and details were not documented due to the patient's and her family's lack of knowledge regarding the specific details of the medications used. However, in similar instances, it may be feasible to establish communication, if possible, with the primary healthcare provider to obtain details that could shed light on the underlying cause of the recurrence and inform future management strategies.

Autoimmune and autoinflammatory diseases, including autoimmune encephalitis, can be considered if the patient has delirium, psychosis, cognitive decline, memory impairment, behavioral deterioration, seizures, and movement abnormalities. One of the differentials that can also be considered is Behcet's disease, which presents with recurrent oral and/or genital ulcers [7]. In case of suspicion of autoimmune disease, investigations may include electroencephalogram (EEG), neuroimaging, neuronal antibody testing, thyroid antibodies, and paraneoplastic evaluation. If there is an MRI/EEG abnormalities or encephalopathic symptoms, lumbar puncture (LP) may be considered [7]. ANA test screening should be considered if PANS criteria are met with high ESR, CRP, cytopenia, or dry eyes and mouth. Antiphospholipid (APL) antibodies should be considered if PANS criteria are met with thrombocytopenia, petechiae, chorea, thrombosis, migraines, stroke, or livedo reticularis [7].

Treatment of PANDAS that were reported in the literature include psychiatric and behavioral interventions (CBT and selective serotonin reuptake inhibitors), antibiotic therapy, NSAIDs, immunomodulatory therapy (steroid, intravenous immunoglobin therapy), and tonsillectomy [2]. Psychiatric and behavioral interventions are conventional interventions that provide direct symptomatic relief and are considered the mainstay of treatment for the behavioral manifestations of PANS. However, the evidence to treat PANS with SSRI is limited to the clinical experience, with no controlled trial done yet [14]. As children with PANDAS may be susceptible to the side effects of serotonin reuptake inhibitors and other medications, “starting low and going slow” is essential when administering these drugs [3]. SSRI may help restrict eating and/or fluid if it is related to OCD. On the other hand, CBT, including exposure and response prevention (ERP) and parent management training (PMT) evidence, is limited to pilot studies with no large-scale studies. CBT also may be used to restrict food or fluid intake and for separation anxiety. Occupation therapy can be considered for patients afraid of choking or vomiting [14].

Immunomodulatory therapy use is controversial. The rationale for use with PANS includes evidence of neurological changes by various investigations, including MRI, positron emission tomography (PET), and psychological assessment. Furthermore, it was reported that patients with PANS have a 70% family history of autoimmune disease or inflammatory disorders. There were two clinical trials regarding using immunomodulatory therapy for PANS. In 1999, double-blind, placebo-controlled (DBPC) showed IVIG and therapeutic plasma exchange (TPE) effective in reducing symptoms in PANDAS by 45%–58% [15]. In 2016, a double-blind, randomized controlled trial showed no statistically significant difference at 6 weeks, but open-label IVIG infusion showed improvement of OCD by 50% at 6 weeks [16]. On the other hand, there are many rationales against using immunomodulatory therapy. Given the elevated incidence of Group A streptococcus (GAS) infection, carriage, and OCD, establishing a definitive causal relationship between these factors is challenging since it remains uncertain if the observed correlation is indeed causative or merely coincidental. Hence, the cooccurrence of OCD and streptococcus infection may be coincidental rather than streptococcus directly inducing OCD via an autoimmune mechanism. Moreover, some studies could not distinguish patients with PANDAS from control based on autoantibody profiles. Furthermore, a longitudinal controlled study reported the failure of immunological markers to correlate with clinical worsening of PANDAS. Also, a study in rodents showed the failure of micro-infusion of PANDAS serum into rodents' stratum to produce changes in behavior. As a result, some advised using caution and avoiding routine immunomodulatory therapy [2].

The evidence of antibiotic treatment for PANDAS is limited. No RCT for children suspected of having PANDAS syndrome [2]. However, four reported cases showed marked improvement in symptoms of anorexia nervosa [14]. There is less evidence for tonsillectomy to treat PANDAS; a systemic review of five studies showed no clear evidence for treating PANDAS with tonsillectomy [2].

In a recent systematic review conducted in 2021, randomized controlled trials were examined to evaluate the efficacy of anti-inflammatory, antibacterial, or immunomodulating treatment [3]. The findings of this research revealed a notable lack of certainty regarding the therapeutic benefits of such treatments. At the same time, there was a reasonable level of certainty regarding adverse effects [3].

There are areas of agreement regarding the controversy of PANDAS. GAS is one of the factors that can exacerbate OCD. Children with signs and symptoms of GAS infections should be evaluated for GAS infection. Children with infection and OCD require standard treatment. Routine administration of immunomodulatory therapy (steroids, plasma exchange, IVIG) is not indicated for children who meet PANDAS. Routine administration of prophylactic antibiotics is not indicated for children who meet PANDAS criteria [2].

## 4. Conclusion

PANDAS is a subtype of PANS with a controversial diagnosis. Criteria for PANS include sudden onset OCD/restricted eating with two emotional, behavioral, cognitive, and neurological symptoms. There are many causes for PANS, including infections and autoimmune diseases. There is limited evidence for the treatment options. SSRI and psychotherapy are considered the main treatment with prompt infection treatment in the case of PANDAS.

## Data Availability

No data are available for this study.
